# Olfactory shifts linked to postpartum depression

**DOI:** 10.1038/s41598-021-94556-z

**Published:** 2021-07-22

**Authors:** Mei Peng, Hazel Potterton, Joanna Ting Wai Chu, Paul Glue

**Affiliations:** 1 Sensory Neuroscience Laboratory, Department of Food Science, University of Otago, Dunedin, New Zealand; 2 National Institute for Health Innovation, Faculty of Medical and Health Science, The University of Auckland, Auckland, New Zealand; 3 Department of Psychological Medicine, School of Medicine, University of Otago, Dunedin, New Zealand

**Keywords:** Depression, Human behaviour

## Abstract

Postpartum Depression (PPD) is the most common non-obstetric complications associated with childbearing, but currently has poor diagnostic regimes. Sensory symptoms of PPD are understudied, particularly with regard to the sense of olfaction. The present study addresses this research gap by assessing differences in olfactory abilities between 39 depressed mothers, who were within the perinatal period (i.e., during pregnancy and up to 1-year post pregnancy) and assessed with Edinburgh Postnatal Depression Scale, and their case-matched healthy volunteers. The assessments include two olfactory testing sessions conducted 4-weeks apart, each comprising a standard odour detection threshold test (i.e., Snap & Sniff Olfactory Test System), and intensity and valence ratings for 3 “pleasant” and 3 “unpleasant” odorants. The results revealed no difference between patients (M = 5.6; SE = 0.3) and control group (M = 5.7; SE = 0.4) in terms of olfactory detection threshold. However, the patients group perceived the 3 “unpleasant” odours as significantly less pleasant (*p* < 0.05), and 2 odorants (1 “pleasant” and 1 “unpleasant”) as less intense. Additionally, these results did not appear to be significantly interacted with the individual’s perinatal stage. The present study is the first to evaluate associations between olfactory function and PPD. Findings from the study suggest that, while PPD has little effect on the early stages of olfactory processing, these conditions may have stronger influence on higher-order olfactory perception, including both hedonic and intensity perception. These novel findings add knowledge to sensory symptoms of PPD.

## Introduction

Postpartum Depression (PPD) represents one of the most common complications associated with childbearing^1, 2^. The DSM-5 includes PPD as a sub-category of the major depressive disorder, which can occur during the pregnancy and postpartum period up to 12 months^3^. The estimated prevalence of PPD is in the range of 6–12% during pregnancy^4, 5^ and up to 20% postpartum^6, 7^. Depression during pregnancy has been linked to increased risk of preterm birth, low birth weight, abnormal foetal heart rate, and delayed intrauterine growth^8, 9^, while depression in the postpartum period can similarly have severe consequences for both mothers and offspring, ranging from maternal sleeping difficulties to delayed/impaired development of cognitive, emotional, verbal and social skills in children. Despite the apparently high prevalence of PPD, diagnosis remains an issue, with recent studies noting that “no more than 20% of the affected women are identified”^1^, calling for more research in this area.

Loss or distortions of sensory perception can be important signs of depressive disorders. An extensive body of literature has linked depression to hypo-sensitivities in vision and hearing^10, 11^. The sense of smell has only recently attracted research attention in relation to mood disorders^12–14^. Indeed, brain networks involved in olfactory function and in the development of depression overlap to a large degree^13^. Specifically, once olfactory information emerges via first-order sensory neurons at the nasal mucosa proximal to the olfactory bulb (OB), it is conveyed to the anterior olfactory nucleus, piriform cortex, and amygdala, which together constitute the primary olfactory cortex. Beyond this cortex, higher-order projections of olfactory information converge on the orbitofrontal cortex, agranular insula, thalamus, hypothalamus, basal ganglia, and hippocampus^13^. A second factor relating the sense of smell to depression is that flavour perception plays a vital role in determining an individual’s appetite and eating behaviour. In particular, loss of appetite and reduced interest in food are common complaints amongst depressed patients, although such symptoms are often overlooked or undermined in clinical assessments due to insufficient testing regimes^15^.

Some recent studies have sought to address the potential link between major depressive disorder (MDD) and shifts in olfactory abilities. These analyses have consistently detected reduced olfactory performance in MDD patients (c.f.,^13^), particularly in the domains of olfactory sensitivity (i.e., odour detection threshold), odour discrimination and identification^12, 16, 17^. By contrast, the effects of MDD on several additional olfactory functions have remained unclear—with some studies reporting no differences in odour hedonic perception^17, 18^, and others suggesting that MDD patients overrate pleasantness of odours^19^. In a systematic evaluation of olfactory changes in MDD^20^, a negative correlation was detected for odour detection sensitivity (i.e., high depression scores associated with low odour sensitivity), but no association with subjective valence and intensity ratings. These authors argued that these contrasting results imply that depression impacts early olfactory processing (i.e., detection), but not higher-order perception.

PPD and MDD share similar symptom profiles, including low moods, anhedonia, restlessness, agitation, and impaired concentration. Clinically, PPD had been considered as a variant of MDD, until recent neurological data provided new insights into the differentiation of PPD and MDD in terms of neurobiological profiles. Specifically, PPD showed decreased activation in amygdala in response to emotional cues, whereas MDD showed increased activation in this area (e.g.,^21, 22^). As a result, a few researchers have recommended consideration of PPD as a separate condition, rather than as the straightforward extension of MDD previously implied under diagnostic classifications^23^. This hypothesis is also supported by emerging psychopharmacological research, in which neurosteroids (e.g., brexanolone) have been reliably effective in treating PPD^24^, but not for MDD (e.g., zuranolone). Food and Drug Administration has recently requested additional clinical trials of zuranolone in depression^25^. Researchers have called for more investigation of clinical differences between PPD and MDD, as resolving the status of these disorders may have important implications for PPD diagnosis, treatment, policy and research^26^.

The present study is the first to assess olfactory abilities in patients with PPD. Findings from the study have two important clinical implications—first, they enhance the current understanding of sensory symptoms of PPD; second, they reveal crucial similarities/differences between PPD and MDD in terms of involvements of olfactory processing. Overall, the study provides important information for assisting diagnosis and prognosis of PPD.

## Methods

### Participants

Thirty-nine PPD females (prenatal N = 18; postnatal N = 21) participated in the current study. Another 39 healthy individuals were matched to the depressed group according to (in diminishing importance): age, perinatal stage, number of previous pregnancies, body mass index (BMI), ethnicity, and socio-economic status.

**Figure 1:**
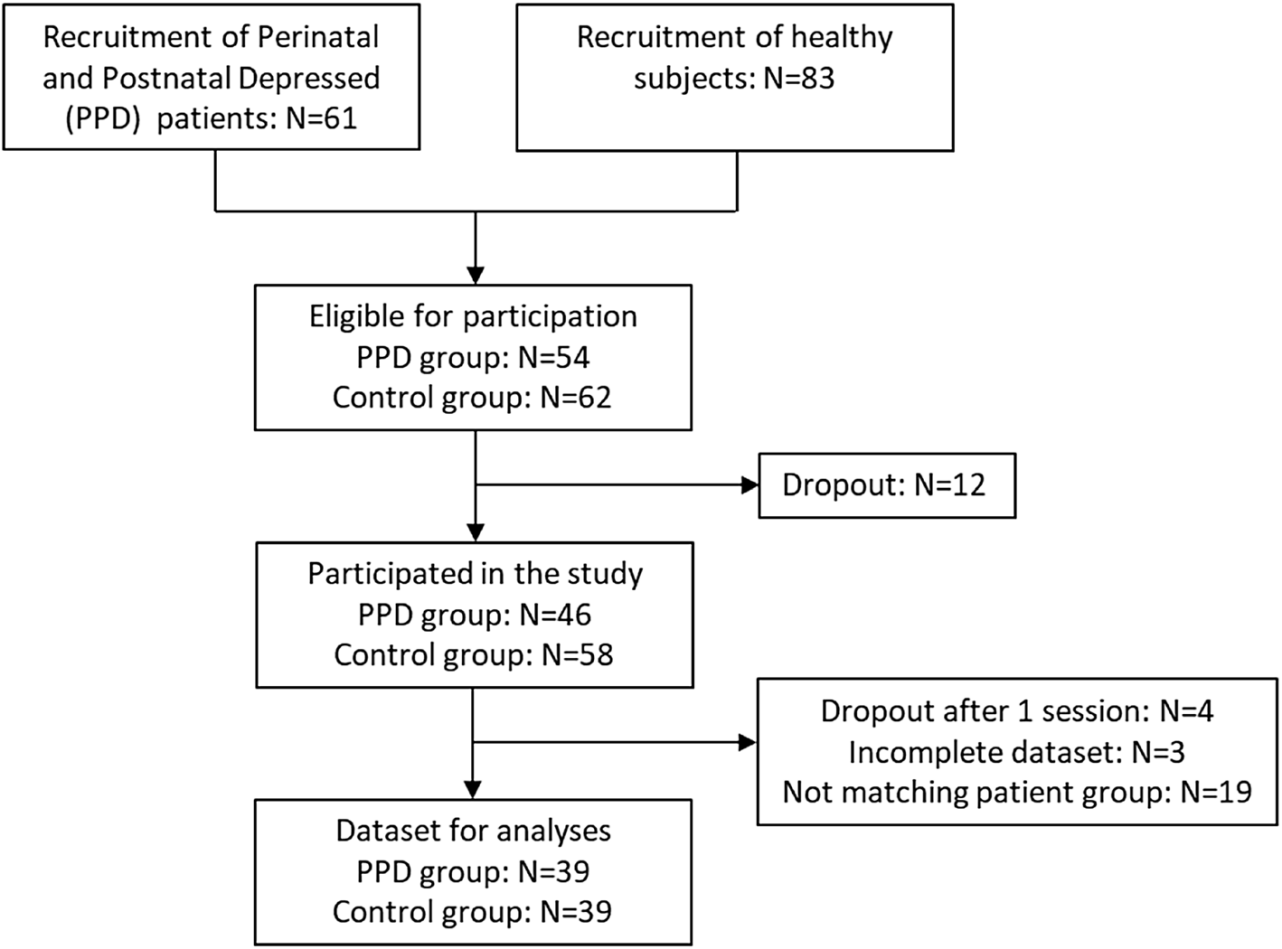
Patient flow diagram.

This study employed convenience and snowball sampling. Recruitment posters were placed in maternity wards, health care clinics, mental health clinics, childcare centres, and throughout the community. Advertisements were also placed in local newspapers and on social media. An open recruiting strategy was used, whereby all potential participants who met the inclusion criteria were invited to participate. A patient diagram is presented in Fig. 1. Notably, a total of 16 participants (out of original 116) dropped out of the study, with 7 of whom from the control group. The primary reason of dropouts given by the participants was lack of time to attend the experiments.

All eligible participants completed the Edinburgh Postnatal Depression Scale before their attendance to the test sessions. All participants gave informed, written consent to participate in the study, in accordance with the Declaration of Helsinki. New Zealand Health and Disability Ethics Committee approved the study (Reference: 18/NTB/127). Monetary compensation was given at the end of the study.

### Study Overview

Each participant attended two 1-h sessions with 4-weeks apart. The study was carried out in a one-to-one setting. Childcare was provided during the testing session in a separate room. Participants were asked to complete three olfactory tests—olfactory threshold test, odour intensity and valence ratings to six odorants—and a series of questionnaire batteries. The Edinburgh Postnatal Depression Scale EPDS;^27^ was used as the primary assessment scale for depression. In accordance to previous research, participants with scores above 10.5 were grouped into the patient group. In addition, all participants completed Hospital Anxiety and Depression Scale^28^ and International Positive and Negative Affect Scale^29^ as confirmatory measures. Demographics were completed at the end of the second session. All questionnaires were developed and presented with Qualtrics (Qualtrics, USA).

### Olfactory threshold testing

Olfactory threshold testing was performed using ‘Snap & Sniff Olfactory Test System’ (Sensonics International, Model: 02,400, Hadden Heights, NJ). The testing kit comprises five blank-odour pens with 15 odorant pens (phenyl ethanol; the concentration is 0.5 log apart). The threshold test employs a staircase testing paradigm with a 2-alternative forced choice presentation method. It has been widely used as a clinical diagnostic tool for olfactory dysfunctions, with high levels of sensitivity and specificity^30^. The experimental procedure followed that of previous studies^31^, and individual thresholds were estimated by averaging the last four reversal points^30^. Notably, the best-estimate odour threshold can range from 2 to 9, with 9 representing high sensitivity and 2 representing low sensitivity.

### Odour Hedonic Testing

For the odour hedonic and intensity rating tasks, three “pleasant” and three “unpleasant” odours were used (see Table 1). These odorants were selected as they are all common in food and therefore should be similarly familiar to the participants^32^. Odorant compounds (Sigma-Aldrich, USA) at 99% purity, were diluted in distilled water, and presented in 50-ml glass brown bottles. Pilot testing had been performed to determine the appropriate concentration for each odorant.

**Table 1:** Information of the odorants used in the present study.

Odorant no	Chemical name	CAS number	Concentration	Characteristics
1	Cis-3-hexen-1-ol	928–96-1	200 ppm	Green grass and leaves (unpleasant)
2	Amyl Acetate	628–63-7	150 ppm	Pears and apples (pleasant)
3	Isovaleric Acid	503–74-2	196 ppm	Rancid; Parmesan cheese (unpleasant)
4	Ethyl-butyrate	105–54-4	150 ppm	Fruity; Pineapple (pleasant)
5	1-Octen-3-ol	3391–86-4	108 ppm	Mushroom (unpleasant)
6	Citral	5392–40-5	200 ppm	Lemon (pleasant)

For the odour hedonic test, the Labelled Affective Magnitude (LAM) Scale was used. LAM scale is a line scale with 100 units anchored with the phrases “greatest imaginable like”-100.00, and “greatest imaginable dislike”-0.00, at the top and bottom, respectively. The LAM scale consisted 9 intermediate anchors (“like extremely”-87.11, “like very much”-78.06, “like moderately”-68.12, “like slightly”-55.62, “neither like/dislike”-50.00, “dislike slightly”-44.69, “dislike moderately”-34.06, “dislike very much”-22.25, and “dislike extremely”-12.25)^33, 34^. Each participant was asked the following, “Please rate your liking or disliking of the indicated sample”. The experimenter placed the glass bottle containing the odorant approximately 2 cm away from the nose and asked the participant to inhale for 2–3 s. A 30 s inter-stimulus interval was present between the presentations of each odour. Each odour was randomised across all participants and sessions. Participants recorded their hedonic perception of each of the six odours presented on the LAM scale.

### Odour intensity testing

For the odour intensity evaluation, the Labelled Magnitude Scale (LMS) test—a psychophysical scaling method for quantifying intensity perception of sensory stimuli^35, 36^—was used. The LMS is composed of 100 units with seven verbal labels (“no sensation”-0, “barely detectable”-1.4, “weak”-6.1, “moderate”-17.2, “strong”-35.4, “very strong”-53.3, “strongest imaginable”-100). Each participant was asked, “Please rate the strength/intensity of the indicated sample”. Presentation of each odour occurred in an identical manner to the LAM scale. Participants recorded their intensity perception of each of the six odours.

### Data analyses

For odour detection thresholds, a 2*2*2 mixed-model ANOVA was performed, with Session (2 sessions) being the within-subject variable, Group (Patients vs. Control) and Perinatal Stage (Prenatal vs. Postnatal) being between-subject variables. Post-hoc tests based on simple effect tests, with *Bonferroni* corrections, were then employed to disentangle any significant main or interaction effects derived from the ANOVA.

For odour intensity and valence data, two separate mixed-model ANOVAs were performed on the overall datasets. With each ANOVA model, Odorants (O1-O6) and Session (2 repeated sessions) were defined as within-subject variables, whereas Group (Patients vs. Control) and Perinatal Stage (Prenatal vs. Postnatal) were between-subject variables. Post-hoc tests with Bonferroni corrections were used to explain any significant effect derived.

Relationships between olfactory functions and depression were further analysed using Pearson correlation analyses. To control for potential influences of age, BMI, and household income, partial correlations were then calculated. These analyses were repeated for each olfactory measure for each odorant. An alpha level of 5% was implemented for detecting statistical significance. All analyses were performed with SPSS (version 26.0, USA).

## Results

### Participant characteristics

The study included 78 participants with equal numbers of depressed and healthy individuals. Table 2 summarises participant characteristics of each of these four sub-groups. Independent t-test on EPDS scores confirmed significant differences between the patient and control group. No substantial difference was observed between these groups in terms of age, BMI and ethnicity.

**Table 2:** Summary of participant characteristics.

	Prenatal participants		Postnatal participants	
	Depressed (N = 18)	Control (N = 18)	Depressed (N = 21)	Control (N = 21)
**Age (year)**				
Range	19–36	20–41	23–37	24–41
Mean	29.7	31.2	29.7	31.2
Standard deviation	4.61	5.70	5.40	4.30
**Ethnicity**				
New Zealand European	77.78%	88.89%	76.19%	80.95%
Māori	5.56%	0%	4.76%	0%
Asian	16.67%	11.11%	14.29%	14.29%
Others	0%	0%	4.76%	4.76%
**Body-mass-index**				
Mean	31.6	30.56	32.15	29.5
Standard deviation	7.66	6.44	6.58	5.19
EPDS Mean (SE)*	16.7 (1.0)	6.1 (0.6)	16.27 (0.8)	6.10 (0.6)

* Edinburgh Postnatal Depression Scale; SE-standard error.

### Odour detection thresholds

The mixed-model ANOVA did not find significant interaction effect across Group, Session and Perinatal Stage (F_(1,72)_ = 1.6, *p* = 0.210), nor significant main effect for each variable (Perinatal Stage: F_(1,72)_ = 2.3, *p* = 0.133; Session: F_(1,72)_ = 1.04, *p* = 0.311; Group: F_(1,72)_ = 0.02; *p* = 0.888). Table 3 summarises the threshold measures for each participant group in separate sessions.

**Table 3:** Mean (M) and standard error (SE) of odour detection threshold (score range 2–9) of the patient and control groups at different perinatal stages.

	Prenatal				p	Postnatal			
	Patient		Control			Patient		Control	
	M	SE	M	SE		M	SE	M	SE
Session 1	5.5	0.17	5.4	0.30		5.8	0.34	6.1	0.33
Session 2	5.8	0.24	5.0	0.37		5.2	0.30	6.2	0.39

Pearson correlation analyses further confirmed that there was no significant relationship between odour threshold and EPDS score (r = −0.13, *p* = 0.26, n = 78). After contracting for age, BMI, and household income, the correlation between the EPDS score and odour threshold remained non-significant (r = −0.15, *p* = 0.19).

### Odour Intensity Ratings

For the odour intensity data, results from the mixed-model ANOVA did not reveal a significant overall interaction effect (F_(5,72)_ = 0.61, *p* = 0.690). While no significant main effect was observed for Group (F_(1,72)_ = 1.97, *p* = 0.161), a significant interaction effect was found between Group and Odorant (F_(5, 72)_ = 4.9, *p* < 0.001). Post hoc tests indicated significant differences between the depression and control group for the Odorant 2 and Odorant 3. For Odorant 5, the difference was close to reaching statistical significance. Consistently across these three odorants, the patient group gave significantly lower ratings than the control group. Table 4 summarises descriptive statistics and *p*-values derived from post-hoc tests for comparing the ratings between the depressed and control group for individual odorants.

**Table 4:** Mean (M) and standard error (SE) of intensity and hedonic ratings from the patient and control groups for the six testing odorants. Significant p-values derived from the post-hoc tests are in bold (p < 0.05).

	Odour intensity rating						Odour hedonic rating					
	Depressed		control				Depressed		control			
Odorants	M	SE	M	SE	*F*_(1, 72)_	*p*	M	SE	M	SE	*F*_(1, 72)_	*p*
1	2.5	0.24	2.9	0.25	1.21	0.273	4.3	0.22	5.6	0.21	4.12	**0.046**
2	4.6	0.25	5.9	0.27	9.72	**0.003**	6.5	0.27	6.7	0.29	0.05	0.813
3	3.4	0.27	4.6	0.29	8.62	**0.004**	3.6	0.22	5.2	0.26	11.2	** < 0.001**
4	3.5	0.27	3.5	0.26	0.01	0.997	6.3	0.23	6.2	0.28	0.05	0.811
5	4.5	0.26	5.1	0.28	3.91	0.051	4.2	0.24	5.3	0.24	9.71	**0.003**
6	3.5	0.29	3.4	0.3	0.01	0.991	5.8	0.2	5.8	0.21	0.06	0.801

Furthermore, the Perinatal Stage had no significant main effect on the intensity ratings (F_(1, 72)_ = 0.15, *p* = 0.70), a two-way interaction effect due to Group × Perinatal Stage were observed for Odorant 3 (F_(1, 72)_ = 5.25, *p* = 0.025). Post-hoc tests with *Bonferroni* corrections revealed that, for both odorants, the difference between the depression and control group was substantially larger at the postnatal than the prenatal stage.

**Figure 2:**
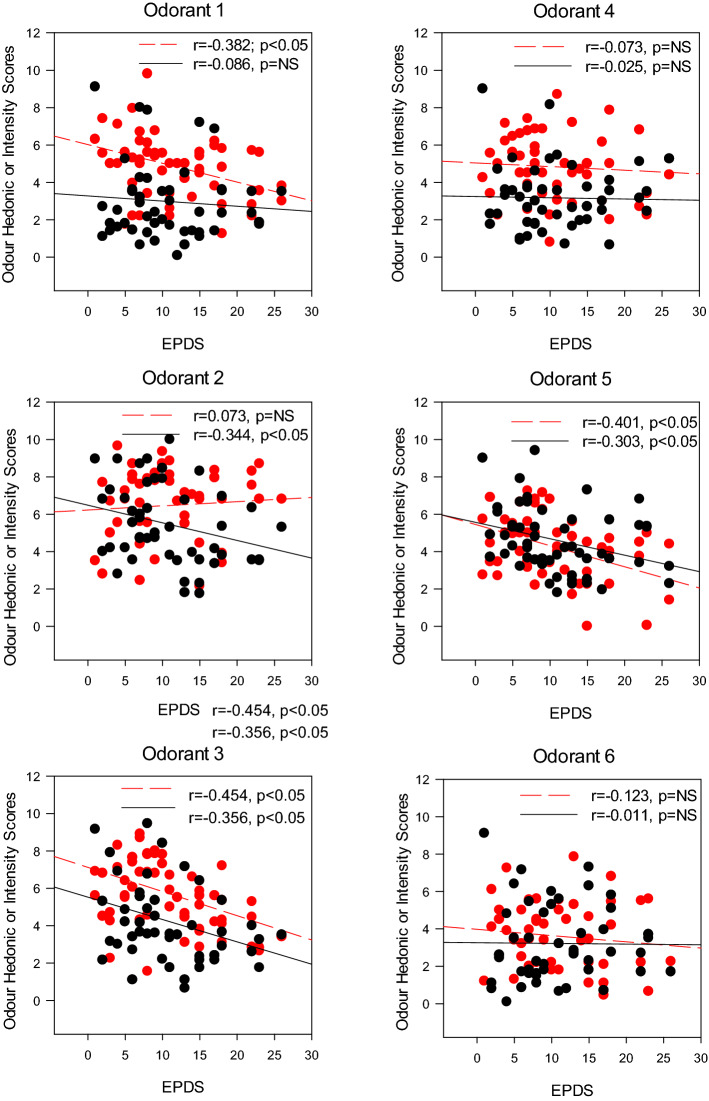
Scatter plots, with simple regression line, illustrating relationships between depression scores (based on Edinburgh Postnatal Depression Scale) and self-reported ratings of intensity (black) and valence (red) to six testing odorants.

Pearson correlation analyses further confirmed a negative relationship between EPDS and intensity ratings for Odorant 2 (r = −0.34; *p* = 0.002), Odorant 3 (r = −0.32; *p* = 0.004), and Odorant 5 (r = −0.30; *p* = 0.008; n = 78)—individuals with higher EPDS rated the odour intensities to be lower. These results were similar after controlling age, BMI and income groups using a partial correlation model. The correlation coefficient was slightly lowered (see Fig. 2). No significant correlation was found for other odorants.

### Odour hedonic ratings

Results from the mixed-model ANOVA of the odour hedonic data did not observe significant interaction effect (F_(5,72)_ = 0.48, *p* = 0.79). Across all independent variables, only Odorant showed a significant main effect (F_(5, 72)_ = 35.7, *p* < 0.001). Furthermore, a significant 3-way interaction was evident for Odorant × Group × Perinatal Stage (F_(5,72)_ = 5.74, *p* = 0.004). Post-hoc tests with *Bonferroni* corrections indicated that the prenatal, depressed group rated Odorant 1 significantly lower than the control group (depressed group: M = 4.7; SE = 0.35; control group: M = 5.9, SE = 0.28), although such difference did not achieve statistical significance for the postnatal group. Additionally, for Odorant 3 and 5, the post-hoc analyses revealed that the depressed group reported significantly lower ratings than the control group (see Table 4).

Furthermore, Pearson correlation revealed significant negative relationship between EPDS and hedonic ratings for Odorant 1, 3 and 5—all three “unpleasant” odours (O1: r = −0.41, *p* < 0.001; O3: r = −0.44, *p* < 0.001; O5: r = −0.42; *p* < 0.001; n = 78). These negative correlations were maintained with partial correlation. These results suggested that individuals with higher depression scores are more likely to rate these “unpleasant” odours worse (see Fig. 2).

## Discussion

The current study tested for changes in olfactory function related to postpartum depression. A key finding is that individuals with PPD are not different from undepressed participants, in terms of their *ability* to detect odorants, whereas the former group can have different intensity and hedonic perception of some odorants. These findings imply that PPD is associated with alterations in higher-order olfactory perception, but not early-processing of odours.

Although anecdotal and self-reported data often suggest that an individual’s sense of smell becomes heightened during pregnancy and postpartum^37, 38^, previous psychophysical studies have contradicted this hypothesis—with no significant difference found in detection thresholds between pregnant women and non-pregnant controls^39–43^. The present study, using the same method as in another study (i.e.,^39^), similarly did not find any notable difference between prenatal and postnatal groups, nor any temporal change in olfactory detectability. In terms of odour hedonic (valence) perception, previous research has suggested that pregnant women generally find odours to be less pleasant than do control populations, albeit with some variation across odorants and individuals^38, 42, 44^. For postnatal women, there is very little empirical data assessing odour valence perception. In the present study, the prenatal and postnatal participants gave similar odour valence ratings for all 6 testing odorants, suggesting stable olfactory valence perception in this time-course (i.e., during pregnancy to one-year after pregnancy). Similarly, with odour intensity perception, some studies have suggested that pregnant women in the first trimester would rate certain odours as more intense^37^, whereas other studies did not find any such differences between pregnant and control group^38, 39^. Findings from the present study supported the latter view based on comparable intensity ratings between prenatal and postnatal groups.

Due to the paucity of previous olfactory research on PPD, it may seem challenging to contextualise the current findings. However, our results can be interpreted in the light of comparable olfactory research on MDD. Previous studies of olfactory effects of depression have focussed primarily on odour detection thresholds. Although a few studies have reported normal sensitivity in MDD patients, an extensive body of evidence suggests that MDD is associated with declines in olfactory sensitivity (i.e., increased odour detection threshold; c.f.,^13^). Intriguingly, these observed olfactory deficits in sensitivity did not appear to affect MDD patients’ odour intensity perception^20^. These findings prompted the authors to hypothesise that MDD affects the early processing of olfactory information, but not higher-order perception. In contrast with previous MDD findings, the present PPD study did not detect changes in olfactory sensitivity, but rather observed some decline in odour valence and intensity perception. These results imply that PPD affects olfactory processing at higher order. Future studies are warranted to differentiate the neural circuitry associated with PPD and MDD, in particular, their distinct involvements of olfactory pathways.

Research on olfactory hedonic perception has particular importance for understanding depression, as this perception is closely linked to anhedonia—a common symptom and major criterion for depression diagnosis^17^. Specifically, pleasantness rating is a feature of the orbitofrontal representation that can be modulated by affective states. Dysfunctions in the prefrontal cortex, as related to depression, are thus hypothesized to have a direct impact on pleasantness ratings. To date, most sensory studies of MDD patients suggest that depressed patients perceive “unpleasant” odours to be significantly worse than healthy controls^45^. However, research using “pleasant” odours has produced mixed results, with some observing differences between depressed and healthy individuals^45^, but others detecting no such differences^17^. These inconsistent results may be attributable to the use of different testing odorants. Our data revealed a clear negative correlation between depression scores and odorant hedonic ratings for the three “unpleasant” odours, whereas no relationship was found for the “pleasant” odours. These differential results between the “pleasant” and “unpleasant” odorants are interesting, as previous neuroimaging data suggested distinct brain responses to odorants with varying hedonic values^46^. Specifically, pleasant odours can activate the medial orbitofrontal cortex (OFC), whereas the unpleasant odours are shown to activate the lateral OFC. These distinctions should be further analysed in the PPD cohort, perhaps using neuroimaging techniques.

Some limitations of the present study merit discussion. First, odour identification and discrimination were outside the scope of our analyses. Future studies are needed to determine whether effects of PPD on olfactory abilities extend to odour identification and discrimination. Second, a large number of the participants included in the present study fall into the overweight group. Previous research has suggested that obesity is closely related to olfactory function^47^. Although we endeavoured to match the patient and control cases, the present findings need to be interpreted with consideration of potential interactions subject to body weight.

Overall, the present study is the first to test for links between olfactory function and PPD. Our results for PPD reveal normal olfactory sensitivity, but altered intensity and hedonic odour perception in these patients. These findings add novel insights into sensory symptoms of PPD. Future studies are warranted to contrast olfactory functions of PPD and MDD, and identify the exact neurological alterations related to these conditions.
